# Development of a dynamic prediction model with the inclusion of time-dependent inflammatory biomarker enhances recurrence prediction after curative surgery for stage II or III gastric cancer

**DOI:** 10.1093/jjco/hyaf075

**Published:** 2025-05-23

**Authors:** Larbi Aluariachy, Koji Oba, Yutaka Matsuyama, Akihiro Kuroda, Yasuhiro Okumura, Koichi Yagi, Yoko Oshima, Takeo Fukagawa, Hideaki Shimada, Yasuyuki Seto

**Affiliations:** Graduate School of Medicine, International University of Health and Welfare, 4-1-26, Akasaka, Minato-ku, Tokyo 107-8402, Japan; Interfaculty Initiative in Information Studies, the University of Tokyo, 7-3-1 Hongo, Bunkyo-ku, Tokyo 113-8655, Japan; Department of Biostatistics, School of Public Health, Graduate School of Medicine, The University of Tokyo, 7-3-1 Hongo, Bunkyo-ku, Tokyo 113-8655, Japan; Department of Biostatistics, School of Public Health, Graduate School of Medicine, The University of Tokyo, 7-3-1 Hongo, Bunkyo-ku, Tokyo 113-8655, Japan; Department of Gastrointestinal Surgery, Graduate School of Medicine, The University of Tokyo, 7-3-1 Hongo, Bunkyo-ku, Tokyo 113-8655, Japan; Department of Gastrointestinal Surgery, Graduate School of Medicine, The University of Tokyo, 7-3-1 Hongo, Bunkyo-ku, Tokyo 113-8655, Japan; Department of Gastrointestinal Surgery, Graduate School of Medicine, The University of Tokyo, 7-3-1 Hongo, Bunkyo-ku, Tokyo 113-8655, Japan; Division of General and Gastroenterological Surgery, Department of Surgery, Faculty of Medicine, Toho University, 6-11-1 Omori-Nishi, Ota-ku, Tokyo 143-8541, Japan; Department of Surgery, Teikyo University School of Medicine, 2-11-1 Kaga, Itabashi-ku, Tokyo 173-0003, Japan; Division of General and Gastroenterological Surgery, Department of Surgery, Faculty of Medicine, Toho University, 6-11-1 Omori-Nishi, Ota-ku, Tokyo 143-8541, Japan; Department of Gastroenterological Surgery and Clinical Oncology, Graduate School of Medicine, Toho University, 6-11-1 Omori-Nishi, Ota-ku, Tokyo 143-8541, Japan; Gastric Surgery Division, National Cancer Center Hospital, 5-1-1 Tsukiji, Chuo-ku, Tokyo 104-0045, Japan

**Keywords:** gastric cancer, recurrence, dynamic prediction, landmarking, surveillance

## Abstract

**Background:**

Postoperative recurrence prediction models for gastric cancer often rely on preoperative or immediate postoperative data, overlooking time-dependent biomarkers from follow-up visits. By incorporating longitudinal biomarker data through a landmarking approach, this study aims to enhance recurrence risk prediction.

**Methods:**

This multicenter study included patients who underwent curative surgery for stage II–III gastric cancer from January 2010 to December 2016 in three hospitals in Tokyo, Japan. Their demographic, clinical, and biomarker data were collected from medical records. Biomarkers were collected at surgery and 3, 6, 9, and 12 months postoperatively. Three prediction models—baseline model, landmarking 1.0, and landmarking 1.5—were developed and compared in terms of their prediction accuracy using four measures: concordance probability, calibration plot, Kaplan–Meier curves stratified with predicted risk, and Net Reclassification Improvement. The models aimed to predict recurrence within three years after surgery, with predictions made one year postsurgery.

**Results:**

The study included 274 patients with gastric cancer, with 62 (22.6%) events occurring within three years. As a result of the variable selection process, lymphatic venous Invasion (LVI), pathological T (pT) stage, pathological N (pN) stage, and baseline prognostic nutritional index (PNI) were chosen. Additionally, in landmarking 1.0 and 1.5, S1 treatment status and PNI-change were also selected as time-dependent predictors. Landmarking 1.5, which incorporates time-dependent biomarkers until one year postsurgery, showed superior performance to the other models in all four measures.

**Conclusions:**

Prediction models incorporating postoperative information could serve as a decision-making tool in clinical practice to more precisely distinguish between patients with high and low risk of recurrence.

## Teaser

Dynamic prediction models using postoperative data outperformed standard models relying on surgery-time information in predicting gastric cancer recurrence within 3 years, showing superior accuracy across four metrics.

## Introduction

Gastric cancer emerged as the sixth most frequently diagnosed cancer globally in 2020, with cases reaching 1.09 million [1]. Despite significant advances in gastric cancer treatment, a considerable number of patients still experience recurrence [2]. Globally, both the incidence and mortality rates of gastric cancer have been declining over the past few decades. However, reports indicate a trend toward younger onset ages for the disease [3]. Preventing recurrence in younger gastric cancer patients is increasingly vital due to the trend of earlier onset. Early and effective intervention may impact their long-term health and survival [4]. This need is further heightened because patients who experience a recurrence often have significantly poorer overall survival rates [4–6].

Structured postoperative surveillance is conducted following gastric cancer surgery to identify recurrences or secondary cancers promptly and is argued to be essential for improving the prognosis of gastric cancer patients [7]. The Japanese Gastric Cancer Association specifies follow-up schedules according to the cancer stage, generally advising a 5-year follow-up for stage II or III gastric cancer post-R0 resection [8]. At the 10th International Gastric Cancer Congress held in 2013, it was emphasized that postoperative surveillance schedules should be individualized based on each patient’s risk of recurrence, including factors such as cancer stage.

Furthermore, the duration of surveillance was also a topic of discussion [9]. While the Japanese Gastric Cancer Association’s postoperative surveillance schedule achieves some degree of individualization by setting schedules based on cancer stages, it lacks personalization regarding the patient’s condition observed during follow-up visits. This means that despite variations in individual patient conditions postsurgery, the follow-up schedule remains uniformly fixed for a certain period for all patients, not fully embracing a genuinely personalized approach to patient care. Considering these challenges, there is growing interest in using prediction models to tailor surveillance [10], with research efforts focused on identifying biomarkers that could help better predict the risk of recurrence [11].

Using prediction models for recurrence risk is instrumental in implementing early interventions to prevent recurrence. These models serve as valuable tools for identifying patients at higher risk of cancer recurrence, enabling healthcare providers to effectively tailor postoperative monitoring and treatment plans. Research on models for predicting postoperative recurrence of gastric cancer is not abundant, and the models reported often focus specifically on peritoneal recurrence due to the high mortality rate following recurrence [6,12,13]. To date, the models for predicting postoperative recurrence of gastric cancer have primarily been based on information available preoperatively or immediately postoperatively [14,15]. They do not account for the longitudinal patient status information obtained during postoperative surveillance visits. This inability to incorporate time-dependent variables into prediction models constrains their potential application to individualizing postoperative surveillance schedules.

In this study, we improve recurrence prediction by developing a dynamic prediction model based on landmarking that incorporates postoperative biomarker information, reflecting postoperative changes in biomarker levels, and demonstrates enhanced accuracy compared to conventional prediction models based solely on preoperative data [16].

## Patients and methods

### Study design and setting

This was a multicenter retrospective study of patients who underwent curative surgery for stage II or III gastric cancer between 1 January 2010, and 31 December 2016, at The University of Tokyo Hospital, Teikyo University Hospital, and Toho University Omori Medical Center, all in Tokyo, Japan. This study was approved by the University of Tokyo Graduate School of Medicine Ethics Committee (Approval Number: 2022203NI). We provided an opportunity for individuals to decline participation in the study by publishing the opt-out document on the university hospitals’ websites and the website of the Biostatistics Course in the graduate school of medicine.

### Patients

We enrolled patients aged 20 years and older who underwent curative surgery for stage II or III gastric cancer performed during the study period from the three hospitals. The study focused on patients who received S-1 monotherapy as postoperative chemotherapy, excluding those who underwent preoperative chemotherapy. We excluded patients with missing demographic data or biomarker data at the time of surgery. Additionally, patients who underwent proximal gastrectomy were excluded due to the low number of cases. Follow-up was conducted according to the follow-up protocol specified in the Japanese Gastric Cancer Treatment Guidelines. Patients underwent regular follow-up visits at 1, 3, 6, 9, and 12 months postoperatively, followed by every 3 months until 24 months after surgery and subsequently every 6 months until 5 years after surgery. At each visit, clinical assessments, including medical history, physical examination, performance status evaluation, and body weight measurement, were performed. Routine laboratory tests, including complete blood count, biochemical tests, carcinoembryonic antigen, and carbohydrate antigen 19-9, were conducted at each follow-up visit. Computed tomography (CT) was performed every 6 months. Postoperative adjuvant chemotherapy was administered for either 6 months or 1 year. A preliminary data check confirmed that the event occurrence rate for patients at Teikyo University Hospital differed from the other two facilities. Therefore, we developed the model using patient data from the other two facilities, while data from Teikyo University Hospital were used for external validation.

### Predictors

Demographic and clinical data were collected from medical records. This included demographic information such as age, sex, height, and weight, as well as clinical characteristics such as LVI, pT stage, pN stages, histological type, type of surgery, and the presence of postoperative complications. For analysis, the pN stages N3a and N3b were combined into N3, and the pT stage T1 and T2 were grouped together as T1&2. Additionally, tumors located at the esophagogastric junction were categorized with those in the upper stomach.

### Biomarkers

From each patient, we extracted data on the following biomarker components at the time of surgery and 3, 6, 9, and 12 months postoperatively: monocytes (/μl), platelets (/μl), C-reactive protein (CRP) (mg/dl), albumin (g/dl), neutrophils (/μl), and lymphocytes (/μl). We used the most recent measurement values for all collected items at each time point. Missing values were imputed using the last observation carried forward (LOCF) method. The inflammatory response was characterized by increased proportions of neutrophils, monocytes, platelets, and CRP (i.e. inflammatory parameters) and decreased proportions of lymphocytes and albumin (i.e. anti-inflammatory parameters). Consequently, assessing any pair among these six serum markers related to inflammatory and anti-inflammatory responses yields 15 unique combinations. If the paired serum markers fall within the inflammatory or anti-inflammatory categories, their respective values are multiplied for these combinations. In contrast, if the pairing includes one marker from each category, their values are divided, reflecting the interplay between inflammatory and anti-inflammatory processes. In addition, PNI was calculated as albumin +0.005 × lymphocyte count. The 14 combinations mentioned above and the PNI were considered potential biomarkers for inclusion in the prediction models. Due to potential measurement limits, which may have led to some patients having CRP values reported as zero, it was impossible to calculate the lymphocyte-to-CRP ratio for these individuals. Therefore, with this consideration, the lymphocyte-to-CRP ratio was removed from the list of candidate biomarkers.

### Outcome

Patient survival and disease recurrence data were obtained from medical records. Recurrence-free survival (RFS) was defined as the interval from the surgery date to the first identification of disease recurrence, whether local or distant, or death due to any cause. Patients without any such events were censored at their last follow-up. Events occurring beyond the 3-year postoperative interval were treated as censored.

### Development of prediction models

In this study, to predict recurrence or death by three years postoperatively, we considered three types of models: a baseline model using only baseline patients’ information, and dynamic prediction models incorporating changes in biomarkers and treatment status over time using the landmarking methods, specifically landmarking 1.0 [16] and landmarking 1.5 [17]. We used data from the University of Tokyo Hospital and Toho University Omori Medical Center for model development, while data from Teikyo University Hospital were used for external validation.

### Three models in this study

#### Baseline model

The baseline model, a Cox proportional hazards model, included recurrence or death within 3 years postoperatively as the outcome, using only information available at the time of surgery as predictors. The predictors were selected through a variable selection method.

#### Landmarking 1.0 (dynamic prediction model)

Landmarking 1.0 employed landmark analysis to focus on patients at risk 1 year after surgery [16]. It excluded patients who experienced events within the first year. This method used a Cox proportional hazards model, where the change in the biomarker from baseline to the last measurement before the 1-year postoperatively milestone served as a predictor, along with the other variables from the baseline model.

#### Landmarking 1.5 (dynamic prediction model)

Landmarking 1.5 modified landmarking 1.0 by substituting the biomarker with its predicted value at the 1-year postoperative value [17]. The predicted value is obtained through the Best Linear Unbiased Prediction, calculated using the biomarkers observed up to 1 year postoperatively.

### Variable selection

From a clinical standpoint, LVI, pT stage, pN stage, and S1 are crucial predictors due to their strong association with recurrence and were, therefore, mandatorily included in the prediction models. Variable selection was done through a Cox proportional hazards model with postoperative recurrence or death as the outcome. This study performed a landmark analysis at the 1-year postoperative point. Hence, the cases used for variable selection were limited to those patients who remained disease-free for 1 year postoperatively. The following procedures were followed, and the variable selection was carried out.

1. For patients with available biomarker data at baseline, any subsequent missing biomarker values postbaseline will be imputed using the LOCF method.
2. A backward selection process [18] will be conducted using predictors noted above, S1, age *S1 (interaction term), and one of the biomarkers in Table 1 as candidate variables, using the “fastbw” function from the “rms” package in R [19]. The model derived from this selection and its corresponding Akaike Information Criterion (AIC) will be recorded.
3. The procedure described in Step 2 will be repeated, substituting different biomarkers each time. This will be done for all biomarkers listed in Table 1.
4. From the recorded AIC values, the model with the smallest AIC will be selected as the final model.

**Table 1:** Fifteen longitudinal inflammatory biomarkers evaluated in this study.

**Biomarker**	**Definition/Unit**
Neutrophil-to-lymphocyte ratio (NLR)	Neutrophil (/μl)/lymphocyte (/μl)
Lymphocyte-to-monocyte ratio (LMR)	Lymphocyte (/μl)/monocyte (/μl)
Platelet-to-lymphocyte ratio (PLR)	Platelet (/μl)/lymphocyte (/μl)
CRP-to-albumin ratio (CAR)	CRP (mg/dl)/albumin (g/dl)
Prognostic nutritional index (PNI)	Albumin (g/dl) + 0.005 × lymphocyte (/μl)
Neutrophil–albumin ratio (NAR)	Neutrophil (/μl)/albumin (g/dl)
Monocyte–albumin ratio (MAR)	Monocyte (/μl)/albumin (g/dl)
Platelet–albumin ratio (PAR)	Platelet (/μl)/albumin (g/dl)
Neutrophil × monocyte	Neutrophil (/μl) × monocyte (/μl)
Neutrophil × platelet	Neutrophil (/μl) × platelet (/μl)
Neutrophil × CRP	Neutrophil (/μl) × CRP (mg/dl)
Monocyte × platelet	Monocyte (/μl) × platelet (/μl)
Monocyte × CRP	Monocyte (/μl) × CRP (mg/dl)
Platelet × CRP	Platelet (/μl) × CRP (mg/dl)
Lymphocyte × albumin	Lymphocyte (/μl) × albumin (g/dl)

### Model comparison

Four measures were used to compare the predictive performance of the models. Kaplan–Meier curves were drawn by dividing patients into high-, medium-, and low-risk groups based on the tertiles of their predicted risks. This approach visually illustrates the survival probabilities over time for each risk group.

We computed the concordance probability using the model-generated risk scores to assess each model’s ability to distinguish between patient outcomes [20]. The concordance probability, often called the C-statistic or the area under the receiver operating characteristic curve, gauges the model’s discriminative ability. It does so by indicating the fraction of pairs of patients in which the one with the lower estimated risk indeed experiences a more extended period without an event. This measure effectively captures the likelihood that the model correctly predicts which patient is less likely to have an event first among pairs of patients.

Calibration plots were used by dividing patients into quintiles or deciles based on their predicted risks. The model’s calibration was assessed by comparing the number of observed events to the expected events within each group. A plot that aligns with the diagonal line indicates a close match between the predicted probability of events and the actual proportion of events.

Finally, the Net Reclassification Improvement (NRI) was used to quantify the correct reclassification of patients when comparing risk scores predicted by two models [21]. This involves calculating the difference between the proportion of correct and incorrect reclassifications among patients who experienced events and those who did not and then summing these differences. An NRI value above zero indicates an improvement in risk prediction due to adding new predictors.

### Internal validation

The internal validation of the prediction models was conducted using the bootstrap method to estimate their performance in future patients similar to those included in the study. This evaluation involved replicating the entire model development process, including variable selection, estimation of regression coefficients, and calculation of the C-statistic by creating samples of the same size through resampling from the original sample 200 times. The performance of prediction models developed from each bootstrap sample was assessed within the bootstrap and original samples [20].

### External validation

We aimed to validate all models externally using data from Teikyo University Hospital. Following the same procedure as model development, patients with missing biomarker data at the time of surgery were excluded. Subsequent measurements at 3, 6, 9, and 12 months were imputed using the LOCF method. The C-statistic was then calculated to assess the models’ performance.

Data analysis was performed using R version 4.3. The findings of this study were reported according to the Transparent Reporting of a multivariable prediction model for Individual Prognosis Or Diagnosis (TRIPOD) recommendations [22].

### Temporal validation

We used data from three institutions, including Teikyo University Hospital, and conducted a temporal validation by splitting the research period into two halves: the first from 1 January 2010, to 31 December 2013, and the second from 1 January 2014, to 31 December 2016. We applied the constructed model to the data split in this manner and assessed the robustness of the results concerning the timing of surgery by calculating the metrics described in the model comparison section.

## Results

### Patients’ characteristics

**Figure 1:**
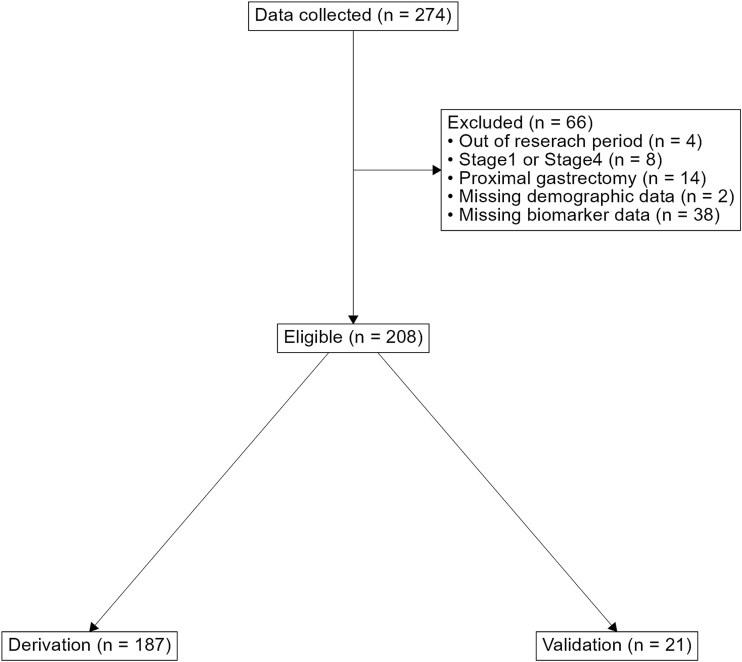
Flow chart.

We collected data from 274 patients who underwent curative surgery for stage II or III gastric cancer. After excluding cases without biomarker measurement at the time of surgery and those with missing demographic data, the analysis included 208 cases. Considering the differences between the University of Tokyo Hospital and Toho University Omori Medical Center compared to Teikyo University Hospital, the former was used as the derivation set, while the latter was used as the validation set (Fig. 1).

Characteristics of patients in the derivation and validation sets were very similar (Table 2). Most patients were elderly (median age 70), predominantly male (derivation: 69%, validation: 81%). Approximately one-third of patients experienced postoperative complications (derivation: 36%, validation: 29%), with the majority undergoing adjuvant chemotherapy for >6 months (derivation: 70%, validation: 67%). LVI was observed in most patients (derivation: 95%, validation: 100%).

**Table 2:** Clinical characteristics of the study population.

**Variable**	**Derivation**, *N* = 187^a^	**Validation**, *N* = 21^a^
Age (years)	70, (25–86)	70, (45–84)
Sex		
Male	129 (69%)	17 (81%)
Female	58 (31%)	4 (19%)
Height (cm)	163, (141–183)	162, (146–175)
Weight (kg)	58, (36–99)	58, (43–71)
Histological type		
Diffuse	91 (49%)	9 (43%)
Intestinal	78 (42%)	12 (57%)
Mixed	18 (9.6%)	0 (0%)
Tumor location		
Upper	39 (21%)	7 (33%)
Middle	72 (39%)	8 (38%)
Lower	64 (34%)	3 (14%)
Whole	7 (3.7%)	3 (14%)
Esophagogastric junction	5 (2.7%)	0 (0%)
Type of surgery		
Distal gastrectomy	106 (57%)	6 (29%)
Total gastrectomy	81 (43%)	15 (71%)
pT stage		
T1	6 (3.2%)	0 (0%)
T2	23 (12%)	4 (19%)
T3	84 (45%)	8 (38%)
T4a	69 (37%)	9 (43%)
T4b	5 (2.7%)	0 (0%)
N stage		
N0	41 (22%)	2 (9.5%)
N1	54 (29%)	5 (24%)
N2	47 (25%)	7 (33%)
N3a	33 (18%)	4 (19%)
N3b	12 (6.4%)	3 (14%)
TNM classification		
IIA	35 (19%)	3 (14%)
IIB	52 (28%)	4 (19%)
IIIA	56 (30%)	7 (33%)
IIIB	34 (18%)	4 (19%)
IIIC	10 (5.3%)	3 (14%)
Lymph node metastasis	2.0, (0.0–28.0)	4.0, (0.0–22.0)
Lymph node dissection	42, (10–96)	36, (13–66)
Lymphatic invasion		
Absent	34 (18%)	2 (9.5%)
Present	153 (82%)	19 (90%)
Venous invasion		
Absent	29 (16%)	3 (14%)
Present	158 (84%)	18 (86%)
LVI		
Absent	9 (4.8%)	0 (0%)
Present	178 (95%)	21 (100%)
Postoperative complications		
Absent	120 (64%)	15 (71%)
Present	67 (36%)	6 (29%)
S1		
Less than 6 months	56 (30%)	7 (33%)
6 months or more	131 (70%)	14 (67%)
Events within 3 years	47 (25%)	15 (71%)

^a^Median, (range); *n* (%).

### Constructed models and their performances

After the variable selection, all models identified LVI, pT stage, pN stage, and baseline-PNI (Table S1). In Landmarking 1.0 and 1.5, S1 treatment status and PNI-change, the change in PNI from baseline, were also selected as time-dependent predictors (Table S2, Table S3).

**Figure 2:**
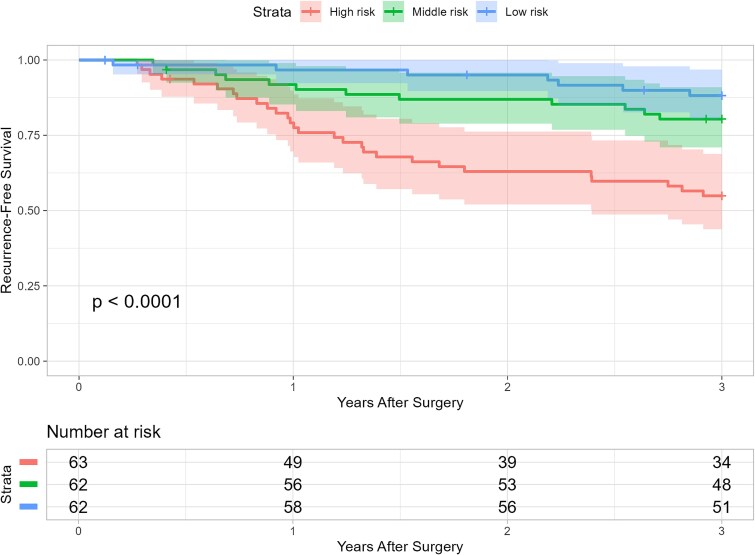
Kaplan–Meier curve of RFS stratified by event risk obtained from the baseline model at 0 year. Patients were classified into three risk groups based on their predicted risk: high risk (<67.0%), middle risk (<86.3%), and low risk (≥86.3%). The *P*-value was obtained from the log-rank test.

A 3-year postoperative recurrence-free survival prediction was conducted at the postoperative point (*n* = 187). Patients at higher risk (<67.0%) demonstrated shorter RFS times from the baseline model (Fig. 2).

**Figure 3:**
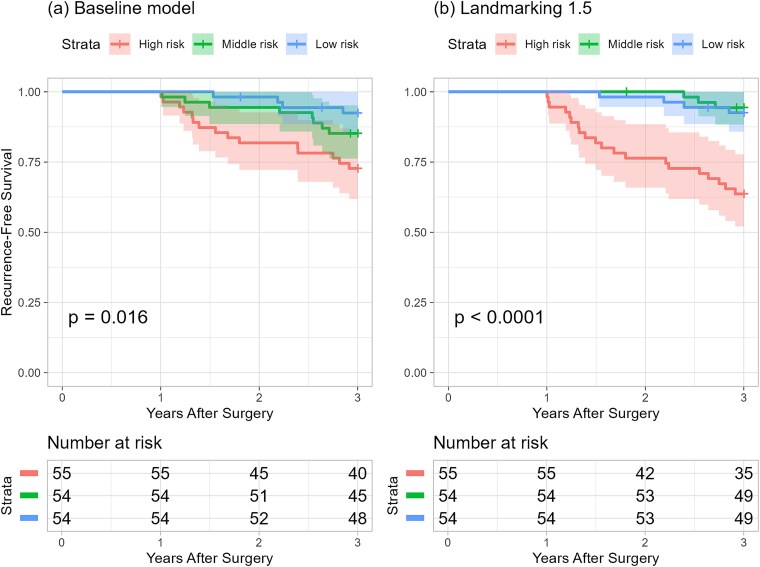
Kaplan–Meier curve of RFS stratified by event risk obtained from (a) baseline model and (b) landmarking 1.5 at 1 year. *P*-values were obtained from the log-rank test. For (a), patients were classified into three risk groups based on their predicted risk: high risk (<67.0%), middle risk (<87.3%), and low risk (≥87.3%). For (b), patients were classified into three risk groups based on their predicted risk: high risk (<86.9%), middle risk (<92.6%), and low risk (≥92.6%). Landmarking 1.5 demonstrates improved predictive accuracy at the 1-year postoperative mark. Landmarking 1.5 better stratifies patients by risk, particularly in identifying high-risk patients.

It was confirmed that using the dynamic prediction models instead of the baseline model improves predictive accuracy when making predictions at the 1-year postoperative mark. Landmarking 1.5 improves on the baseline model in stratifying patients based on predicted risk, especially in identifying high-risk patients (Fig. 3).

Landmarking 1.5 demonstrated superior discriminative ability compared to the baseline model. Using the bootstrap method for internal validation [23], the baseline model achieved an optimism-corrected C-statistic of 0.617. In contrast, Landmarking 1.5 recorded an optimism-corrected C-statistic of 0.671, marking an absolute enhancement of 5.4% (a relative improvement of 8.8%). The 95% bootstrap confidence intervals [24] for the difference in C-statistic between the landmarking 1.5 and baseline models ranged from 0.018 to 0.152. In comparison, the confidence intervals for the difference between landmarking 1.0 and the baseline model ranged from −0.033 to 0.106.

**Figure 4:**
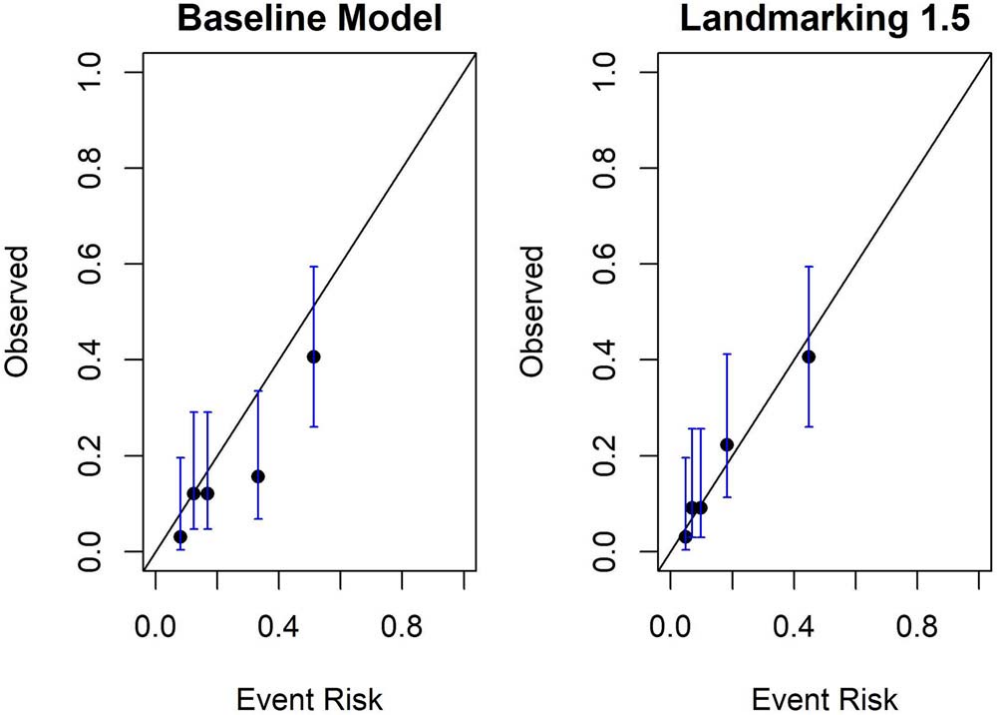
Calibration plot of the prediction models the calibration of the landmarking 1.5 shows superior alignment between predicted and observed outcomes compared to the baseline model.

Figure 4 shows the performance of the model calibration. The calibration of the landmarking 1.5 was superior to that of the baseline model.

Although not statistically significant, the NRI was 0.167 (95% confidence interval, −0.115 to 0.450) and indicated an enhancement in the predictive performance of the landmarking 1.5 compared to the baseline model (Table 3).

**Table 3:** Reclassification table for nonevents and events.

**LM1.5** **Baseline**	**<11.6%**	**11.6%–16.7%**	**16.7%–36.0%**	**≧36.0%**	**Total**
**<11.6%**	36	1	2	0	39
**11.6%–16.7%**	23	11	2	0	36
**16.7%–36.0%**	26	4	1	3	34
**≧36.0%**	7	2	4	14	27
**Total**	92	18	9	17	136
absent					
**LM1.5** **Baseline**	**<11.6%**	**11.6–16.7%**	**16.7–36.0%**	**≧36.0%**	**Total**
**<11.6%**	1	0	1	0	2
**11.6%–16.7%**	3	0	1	0	4
**16.7%–36.0%**	2	2	1	2	7
**≧36.0%**	1	0	3	10	14
**Total**	7	2	6	12	27
Present					

LM1.5, landmarking 1.5; Baseline, baseline model. The upper table represents patients without events, while the lower table represents patients with events. NRI was 0.1672 (95% confidence interval: −0.1151 to 0.4495), demonstrating a potential improvement in the predictive performance of the landmarking 1.5 compared to the baseline model.

We externally validated the prediction models developed using patient data from Teikyo University Hospital. Among patients at Teikyo University Hospital who had complete PNI data at the time of surgery and had not experienced any events by 1 year postoperatively, there were 13 patients. Among these, eight patients experienced events between 1 and 3 years postoperatively. The baseline model, landmarking 1.0, and landmarking 1.5 all demonstrated high discriminative performance, with C-statistics of 0.821, 0.857, and 0.800, respectively.

We performed a temporal validation by evaluating the model’s performance separately for cases from the two time periods: 1 January 2010, to 31 December 2013, and 1 January 2014, to 31 December 2016. The results confirmed that differences in the timing of surgery did not have a significant impact on the model’s performance (Tables S4–S6, Figs. S1–S6).

## Discussion

We evaluated the predictive performance of dynamic prediction models relative to the baseline model, aiming to develop a recurrence prediction model that incorporates time-dependent biomarker information obtained postoperatively. In this study, PNI was selected as a time-dependent biomarker. Landmarking 1.5, incorporating time-dependent PNI related to recurrence, showed improved predictive performance over the baseline model after a comprehensive comparison. These results reflect that the baseline model relied solely on data available at the time of surgery to predict a patient’s recurrence risk 3 years postoperatively, failing to incorporate any postoperative changes in the patient’s condition into the recurrence risk prediction. In contrast, landmarking 1.5 was designed to include postoperative information, enabling it to adjust predictions based on changes in a patient’s health status after surgery.

At the time immediately following surgery, the baseline model was capable of moderately predicting recurrence within 3 years. However, at 1 year postoperatively, landmarking 1.5 outperformed the baseline model in accurately predicting recurrence up to 3 years. Notably, it enhanced the ability to identify patients in the high-risk group (those with a recurrence risk of 63% or higher). By updating the PNI measurements to the values at 1 year postoperatively and reflecting the status of adjuvant S1 therapy, we could capture the condition of patients more precisely. Conversely, because the baseline model did not account for postoperative changes in PNI or the adjuvant S1 therapy status; probably, many patients classified as high risk (with a recurrence risk over 55%) did not subsequently experience an event.

In the study by Spolverato *et al*. [25], using the landmark approach to predict postoperative recurrence of gastric adenocarcinoma, the reported C-statistic was 0.86, indicating a high discriminative ability. The patient cohort in Spolverato *et al*.’s study included those who received preoperative neoadjuvant chemotherapy (17.9%), and approximately half of the patients received postoperative adjuvant chemotherapy (57.2%). In contrast, our study excluded patients who received preoperative chemotherapy and focused solely on those who received postoperative chemotherapy, highlighting the different nature of the target populations. Spolverato *et al*.’s prediction model comprised lymph node dissection, lymph node ratio, histological type, LVI, pT stage, and tumor location. These variables are similar to those in the prediction models developed in this study. A significant difference between their prediction model and ours is the inclusion of time-dependent variables. While their prediction model used only baseline predictors, our model incorporated biomarkers that fluctuate within the first year postoperatively and the status of postoperative adjuvant chemotherapy, representing a novel approach.

By incorporating postoperative data, landmarking 1.5 outperformed the baseline model in predicting recurrence. This dynamic approach allows surveillance to be adjusted based on recurrence risk, minimizing unnecessary visits for lower-risk patients and intensifying follow-up for those at higher risk. Early detection of recurrence provides a crucial chance for effective intervention, especially when patients are still fit enough to receive surgical or medical therapy [4], which is expected to lead to prolonged survival. Furthermore, reducing visits for low-risk patients is beneficial from a cost-effectiveness perspective, a topic under discussion in the literature [26].

In our external validation using data from Teikyo University Hospital, all prediction models demonstrated discriminative solid performance, with a C-statistic exceeding 0.8 across the baseline model, landmarking 1.0, and landmarking 1.5 models. These results indicate a high level of accuracy in distinguishing between patients at varying levels of recurrence risk. However, the small sample size of the external dataset necessitates caution. While these findings are promising, they underline the importance of further validation studies. Such studies ideally involve more extensive, diverse patient cohorts to ensure the robustness and generalizability of the developed models. This step is crucial for affirming the model’s applicability in clinical settings and its potential impact on patient management and outcomes.

The landmark analysis presented here is limited by the reduced sample size at the landmark time point, an issue exacerbated in smaller study populations. While additional landmark points beyond 1 year postsurgery are needed to establish surveillance schedules based on dynamic prediction, this requires larger cohorts for further investigation. Crucially, since S-1 was the standard adjuvant therapy for stage III gastric cancer when these data were collected but is no longer the standard of care, further validation with a contemporary cohort receiving current standard treatment is necessary to confirm the applicability of these findings to current clinical practice. Longitudinal data studies face the issue of missing values. In this study, patients with missing baseline biomarkers were excluded, and later, missing values were imputed using LOCF. This method might not accurately reflect the true changes in biomarkers over time. The internal validation process must fully replicate the model construction procedure. Ideally, resampling should involve repeating level mergers based on predictor-level distributions, but this adds complexity, so this study took the defined levels as given. This might lead to levels that need to be more sized in bootstrap samples and impact optimism calculation. Lastly, due to small sample sizes, further validation is needed for external validation of the prediction models.

Our findings highlight the potential of prediction models that incorporate postoperative biomarker information. Such models could serve as valuable decision-making tools in clinical practice, allowing for a more precise distinction between patients at high and low risk of recurrence, thereby improving personalized treatment strategies.

## Supplementary Material

Supplementary_Figure1_hyaf075

Supplementary_Figure2_hyaf075

Supplementary_Figure3_hyaf075

Supplementary_Figure4_hyaf075

Supplementary_Figure5_hyaf075

Supplementary_Figure6_hyaf075

supplementary_rev0325_hyaf075

Supplementary_Table2_hyaf075

Supplementary_Table3_hyaf075

Supplementary_Table4_hyaf075

Supplementary_Table5_hyaf075

Supplementary_Table6_hyaf075
