# Supplementary figures and images for: Development of a dynamic prediction model with the inclusion of time-dependent inflammatory biomarker enhances recurrence prediction after curative surgery for stage II or III gastric cancer
Source: Jpn J Clin Oncol. 2025 May 23;55(8):871–9. doi: 10.1093/jjco/hyaf075 (PMC12319220; doi:10.1093/jjco/hyaf075)

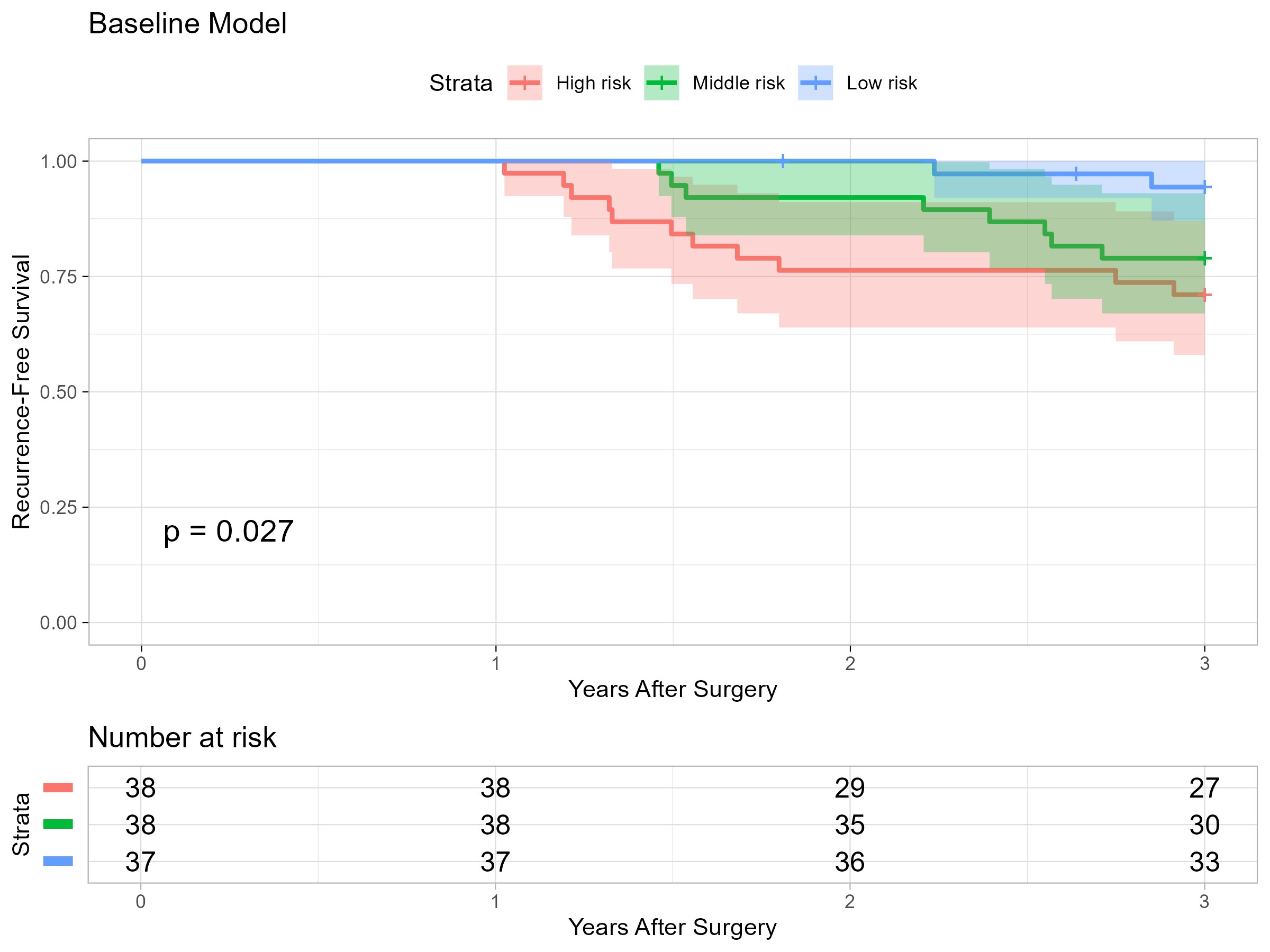

Supplement: Supplementary_Figure1_hyaf075 [file supplementary_figure1_hyaf075.jpeg]

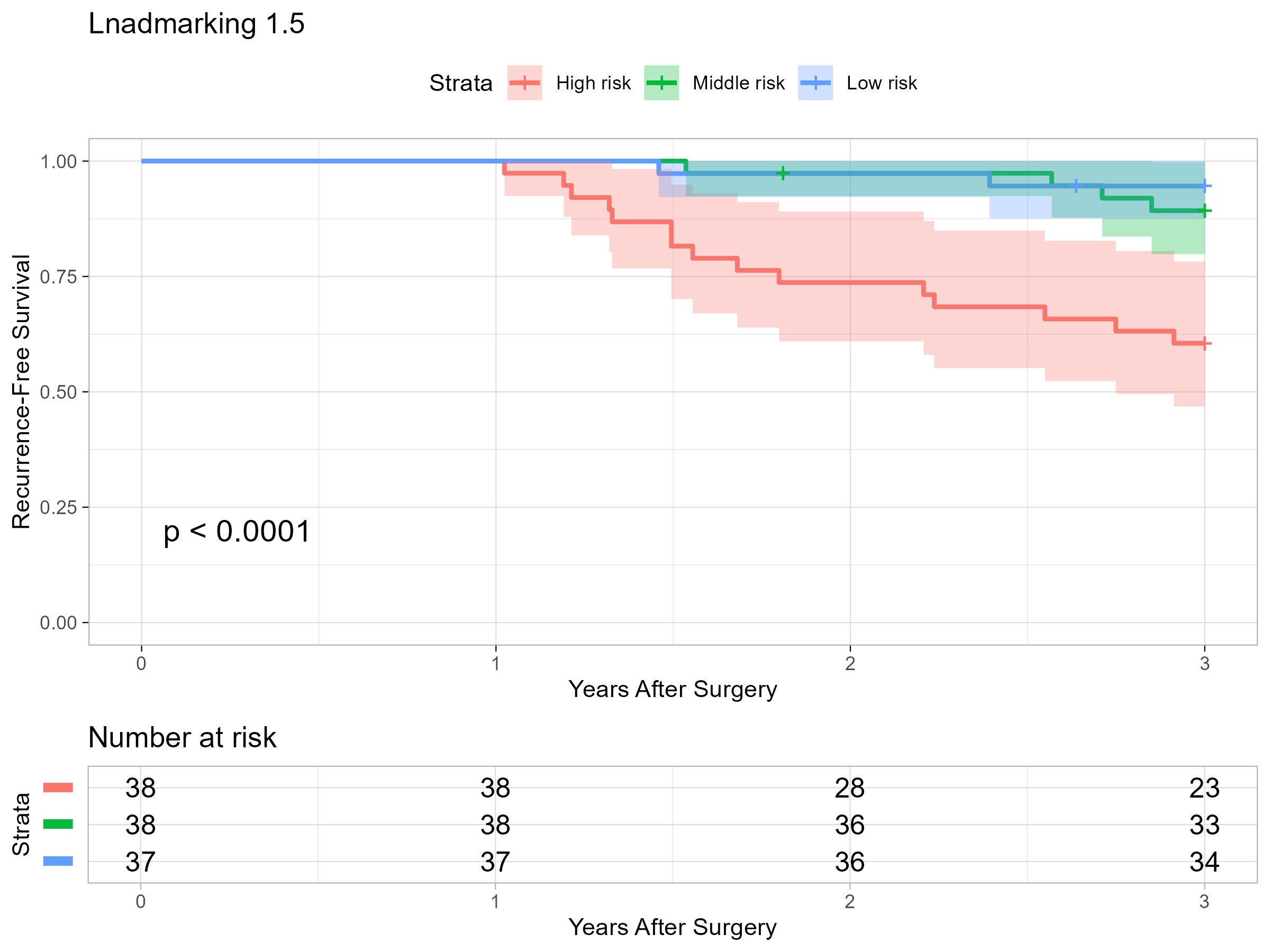

Supplement: Supplementary_Figure2_hyaf075 [file supplementary_figure2_hyaf075.jpeg]

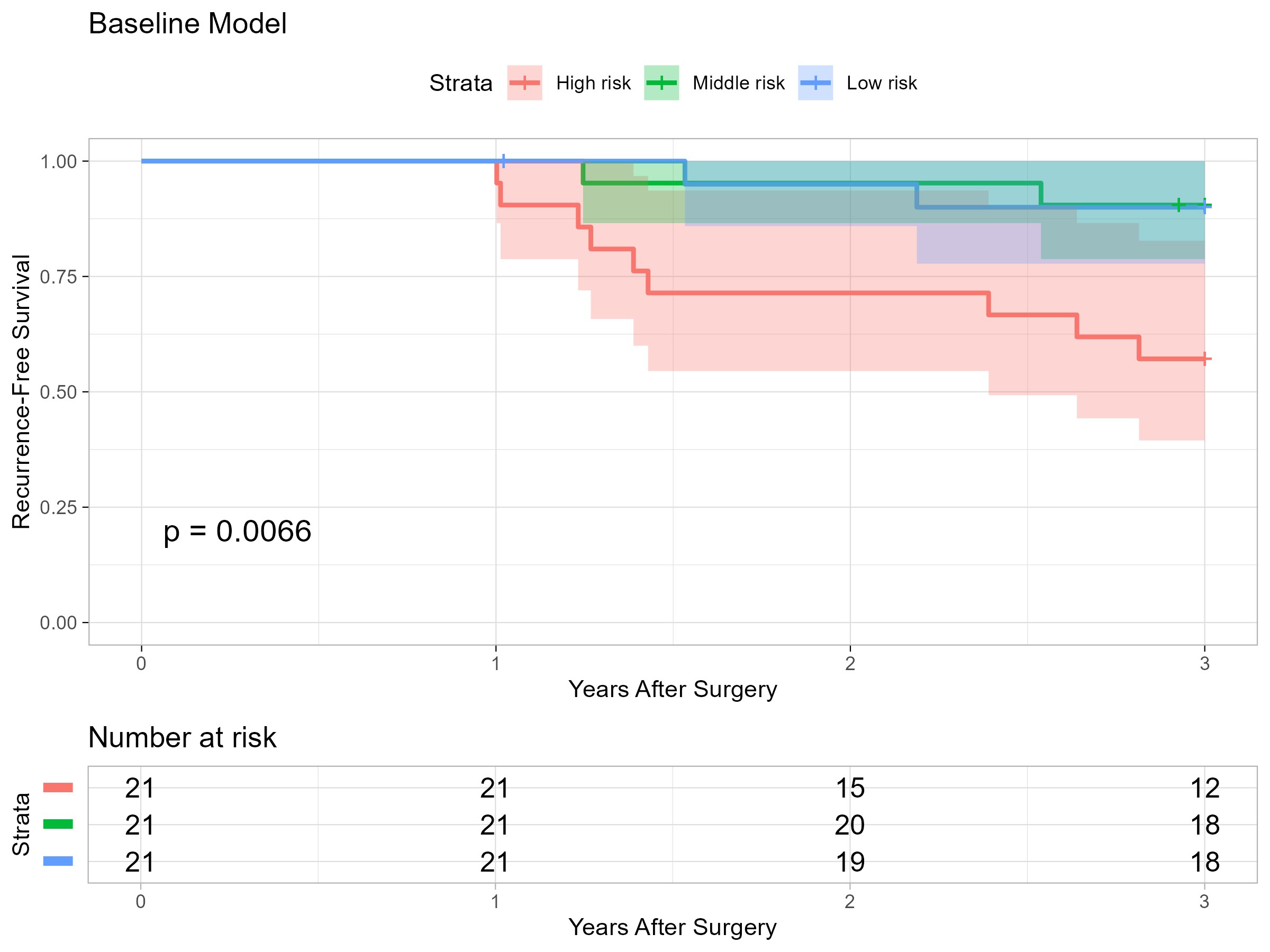

Supplement: Supplementary_Figure3_hyaf075 [file supplementary_figure3_hyaf075.jpeg]

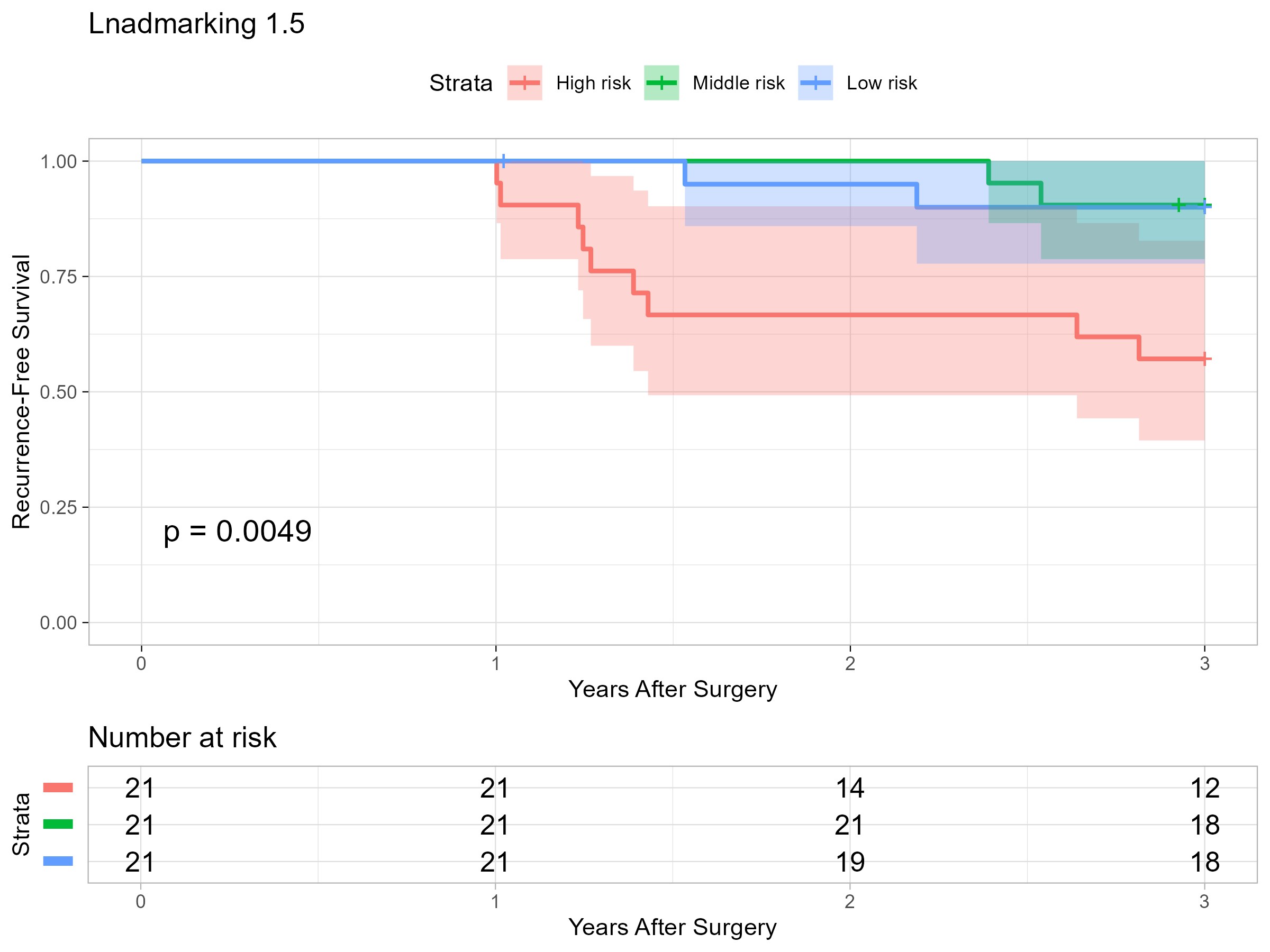

Supplement: Supplementary_Figure4_hyaf075 [file supplementary_figure4_hyaf075.jpeg]

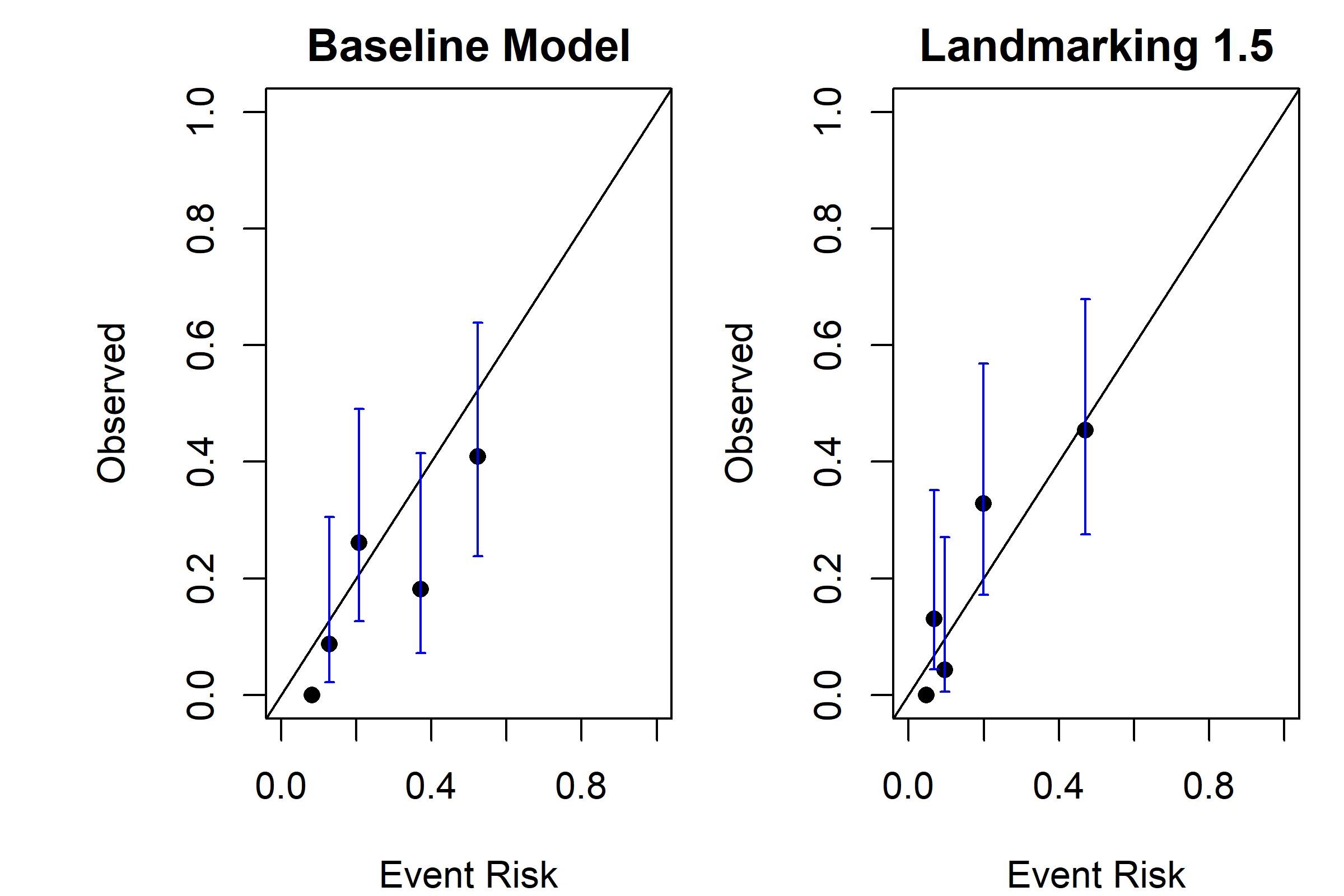

Supplement: Supplementary_Figure5_hyaf075 [file supplementary_figure5_hyaf075.jpeg]

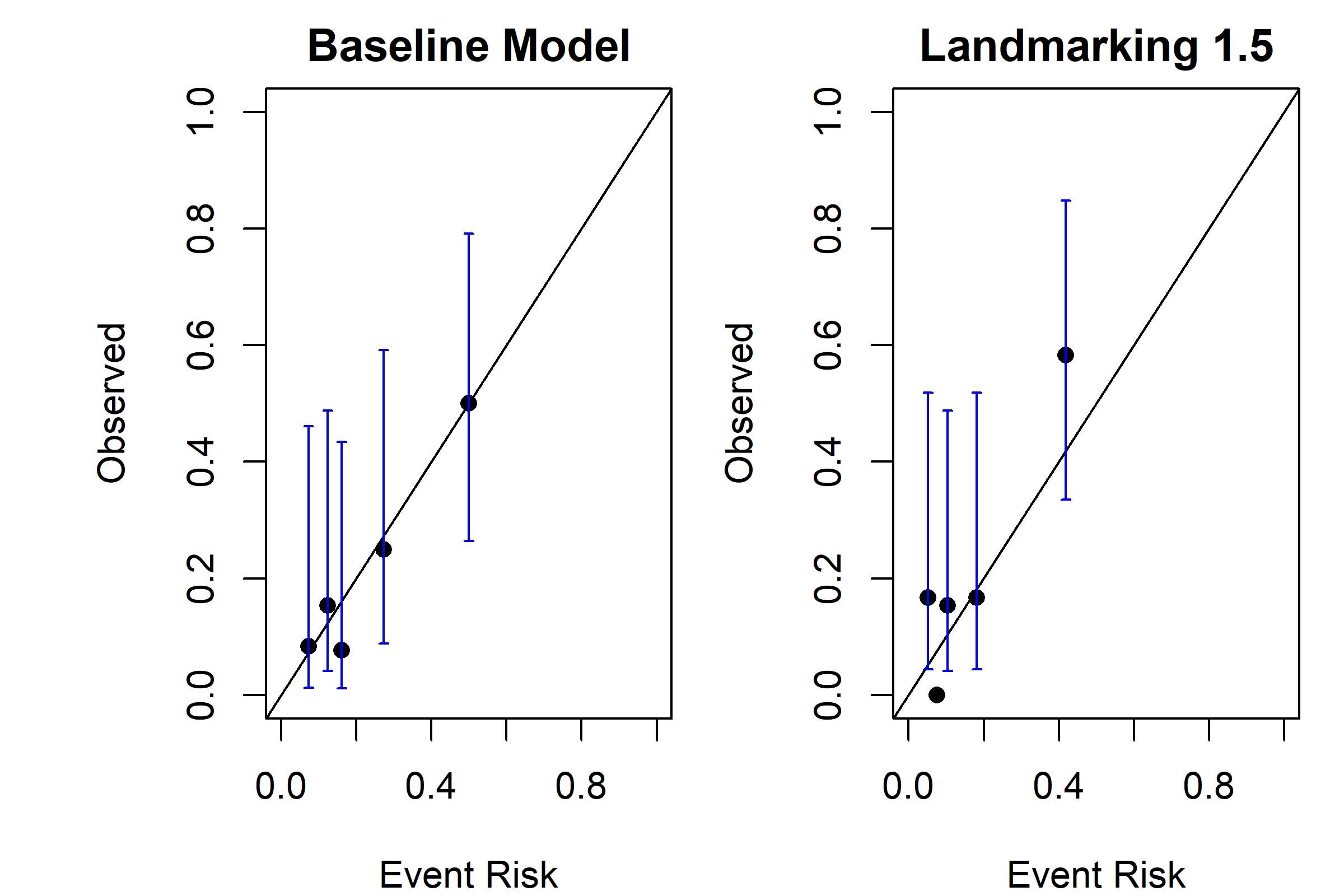

Supplement: Supplementary_Figure6_hyaf075 [file supplementary_figure6_hyaf075.jpeg]
