# Supplementary material for: Development of a dynamic prediction model with the inclusion of time-dependent inflammatory biomarker enhances recurrence prediction after curative surgery for stage II or III gastric cancer
Source: Jpn J Clin Oncol. 2025 May 23;55(8):871–9. doi: 10.1093/jjco/hyaf075 (PMC12319220; doi:10.1093/jjco/hyaf075)
Supplement: supplementary_rev0325_hyaf075 [file supplementary_rev0325_hyaf075.doc]

**SUPPLEMENTAL MATERIALS**

**Baseline model**


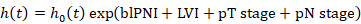


Table S1 Cox regression coefficients for baseline model

|  | Effect | SE | 95% CI | |  |
| --- | --- | --- | --- | --- | --- |
| blPNI | -0.001 | 0.213 | -0.419 | 0.417 |  |
| LVI...YES.NO | 0.503 | 1.029 | -1.514 | 2.519 |  |
| pT stage...T3.T12 | 0.741 | 0.550 | -0.338 | 1.819 |  |
| pT stage...T4.T12 | 0.952 | 0.550 | -0.127 | 2.031 |  |
| pN stage...N1.N0 | -0.296 | 0.620 | -1.510 | 0.919 |  |
| pN stage...N2.N0 | 0.998 | 0.498 | 0.021 | 1.974 |  |
| pN stage...N3.N0 | 1.480 | 0.470 | 0.558 | 2.402 |  |
| 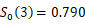 | | | | | |


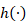
: hazard function


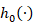
: baseline hazard function


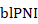
: PNI measured at baseline

CI: confidence interval

SE: standard error


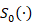
: baseline survival function

**Landmarking 1.0**


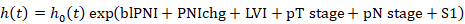


Table S2 Cox regression coefficients for landmarking 1.0

|  | Effect | SE | 95% CI | |
| --- | --- | --- | --- | --- |
| blPNI | 0.264 | 0.421 | -0.561 | 1.088 |
| PNIchg | 0.203 | 0.424 | -0.628 | 1.034 |
| LVI...YES.NO | -0.192 | 1.062 | -2.273 | 1.889 |
| pT stage...T3.T12 | -0.083 | 0.619 | -1.296 | 1.131 |
| pT stage...T4.T12 | 0.278 | 0.605 | -0.907 | 1.464 |
| pN stage...N1.N0 | -0.787 | 0.770 | -2.296 | 0.722 |
| pN stage...N2.N0 | -0.413 | 0.760 | -1.902 | 1.076 |
| pN stage...N3.N0 | 1.455 | 0.548 | 0.381 | 2.529 |
| S1...6 months or more. less than 6 months | -0.162 | 0.485 | -1.113 | 0.789 |
| 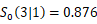 | | | | |


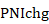
: Change in PNI from baseline

**Landmarking 1.5**

We fitted a Gaussian process to the data, assuming that change in PNI from baseline has a mean function
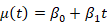
. The covariance function
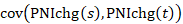
 was assumed to have the structure
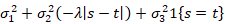
, incorporating a variance
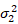
 within subjects, an exponential decay parameter
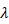
, and a random error
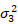
.


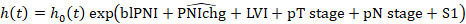


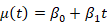


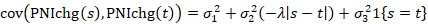


Table S3 Cox regression coefficients for Landmarking 1.5

|  | Effect | SE | 95% CI | |
| --- | --- | --- | --- | --- |
| blPNI | 0.439 | 0.451 | -0.444 | 1.323 |
| 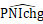 | 0.415 | 0.457 | -0.479 | 1.310 |
| LVI...YES.NO | -0.237 | 1.063 | -2.321 | 1.847 |
| pT stage...T3.T12 | -0.099 | 0.619 | -1.312 | 1.114 |
| pT stage...T4.T12 | 0.297 | 0.604 | -0.887 | 1.480 |
| pN stage...N1.N0 | -0.786 | 0.771 | -2.297 | 0.725 |
| pN stage...N2.N0 | -0.389 | 0.763 | -1.884 | 1.106 |
| pN stage...N3.N0 | 1.499 | 0.552 | 0.417 | 2.580 |
| S1...6 months or more.  less than 6 months | -0.130 | 0.489 | -1.088 | 0.829 |
| 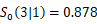  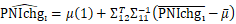  　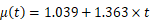  　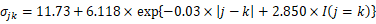 | | | | |


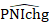
: Predicted change in PNI from baseline


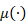
: mean of the linear model fitted to the change in PNI from baseline


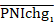
: observed history of PNI change from baseline for subject
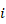
 up to before the landmark time
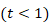
, namely
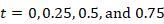


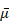
:
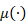
 evaluated at the observed measurement time points up to before the landmark time
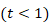


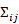
:
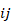
-th submatrix of the variance-covariance matrix for the change in PNI from baseline, with its
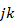
-th component givin by
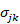


**Temporal validation**

Table S4 C-statistic for temporal validation

|  | 2010-01-01 to 2013-12-31 | 2014-01-01 to 2016-12-31 |
| --- | --- | --- |
| Baseline model | 0.716 | 0.729 |
| Landmarking 1.0 | 0.782 | 0.717 |
| Landmarking 1.5 | 0.772 | 0.732 |

Table S5 Reclassification table for nonevents and events based on data from 2010-01-01 to 2013-12-31

| **LM1.5**  **Baseline** | **<12.7%** | **12.7-17.4%** | **17.4-39.0%** | **≧39.0%** | **Total** |
| --- | --- | --- | --- | --- | --- |
| **<11.6%** | 26 | 0 | 1 | 0 | 27 |
| **11.6-16.7%** | 17 | 5 | 1 | 0 | 23 |
| **16.7-36.0%** | 22 | 0 | 1 | 1 | 24 |
| **≧36.0%** | 4 | 0 | 5 | 9 | 18 |
| **Total** | 69 | 5 | 8 | 10 | 92 |

Absent

| **LM1.5**  **Baseline** | **<12.7%** | **12.7-17.4%** | **17.4-39.0%** | **≧39.0%** | **Total** |
| --- | --- | --- | --- | --- | --- |
| **<11.6%** | 0 | 1 | 0 | 0 | 1 |
| **11.6-16.7%** | 3 | 1 | 1 | 0 | 5 |
| **16.7-36.0%** | 3 | 0 | 0 | 1 | 4 |
| **≧36.0%** | 0 | 0 | 2 | 9 | 11 |
| **Total** | 6 | 2 | 3 | 10 | 21 |

Present

NRI [95% CI]: 0.251 [ -0.063 - 0.5651 ] ; p-value: 0.11715

**Table S6 Reclassification table for nonevents and events based on data from 2014-01-01 to 2016-12-31**

| **LM1.5**  **Baseline** | **<12.7%** | **12.7-17.4%** | **17.4-39.0%** | **≧39.0%** | **Total** |
| --- | --- | --- | --- | --- | --- |
| **<11.6%** | 13 | 2 | 0 | 0 | 15 |
| **11.6-16.7%** | 8 | 4 | 1 | 0 | 13 |
| **16.7-36.0%** | 3 | 7 | 1 | 2 | 13 |
| **≧36.0%** | 5 | 0 | 0 | 4 | 18 |
| **Total** | 29 | 13 | 2 | 6 | 92 |

Absent

| **LM1.5**  **Baseline** | **<12.7%** | **12.7-17.4%** | **17.4-39.0%** | **≧39.0%** | **Total** |
| --- | --- | --- | --- | --- | --- |
| **<11.6%** | 1 | 0 | 0 | 0 | 1 |
| **11.6-16.7%** | 2 | 0 | 0 | 0 | 2 |
| **16.7-36.0%** | 0 | 1 | 1 | 1 | 3 |
| **≧36.0%** | 1 | 0 | 0 | 6 | 7 |
| **Total** | 4 | 1 | 1 | 7 | 13 |

Present

NRI [95% CI]: 0.1292 [ -0.2327 - 0.4912 ] ; p-value: 0.48402

LM1.5: Landmarking 1.5, Baseline: Baseline model


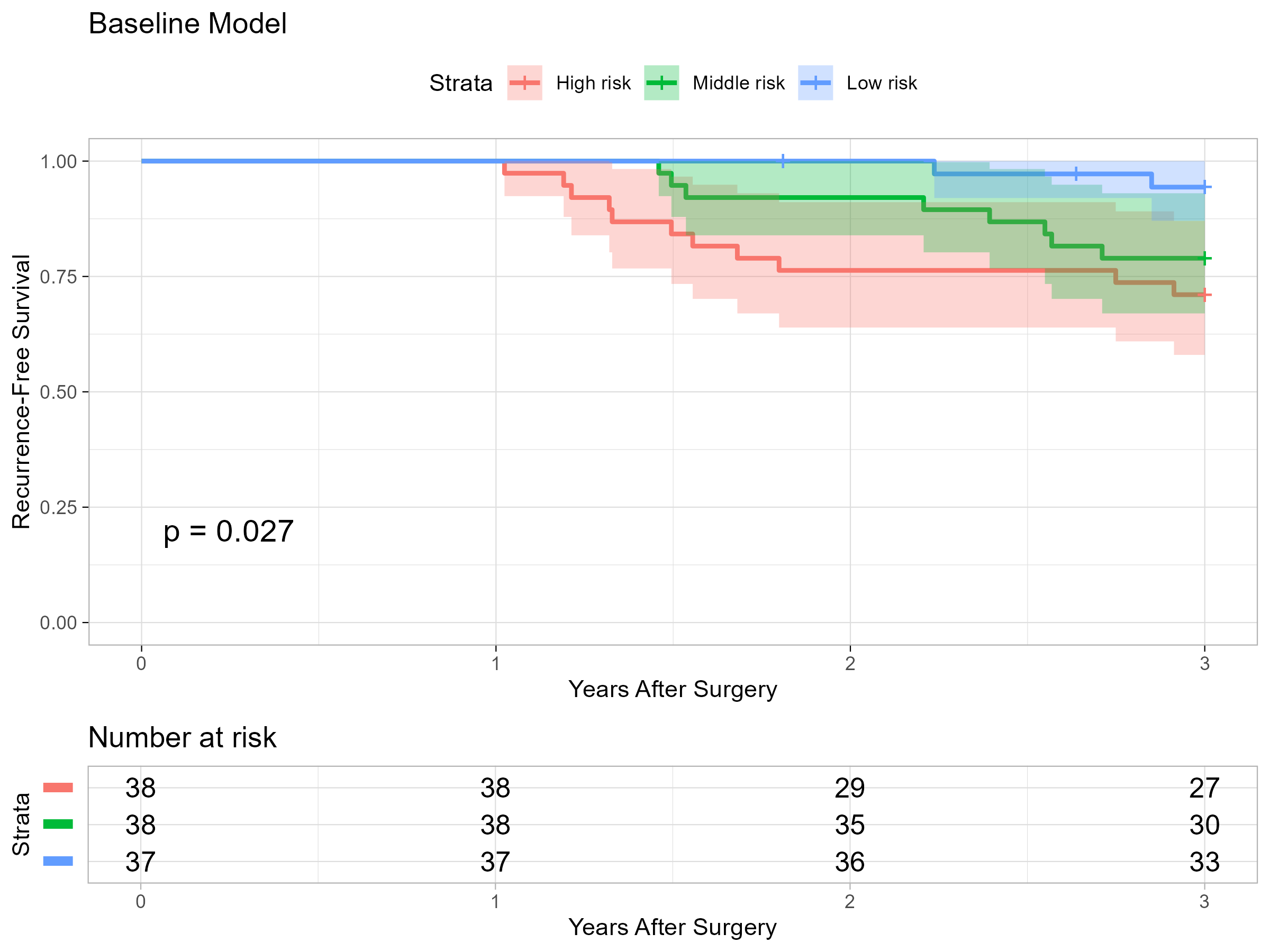


Figure S1 Kaplan-Meier curve of RFS stratified by event risk obtained from baseline model based on the data from 2010-01-01 to 2013-12-31.

**
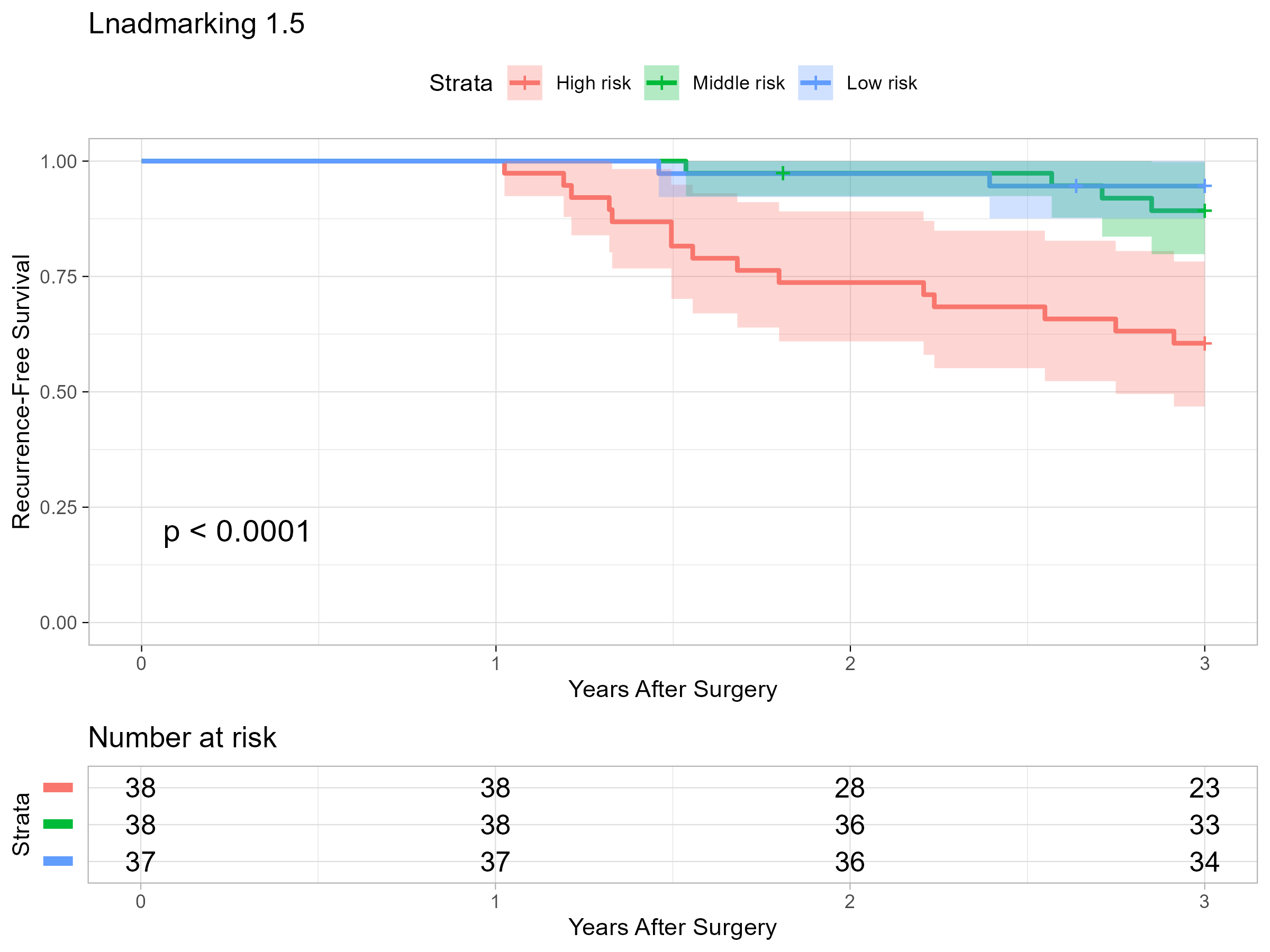
**

Figure S2 Kaplan-Meier curve of RFS stratified by event risk obtained from landmarking 1.5 based on the data from 2010-01-01 to 2013-12-31.

**
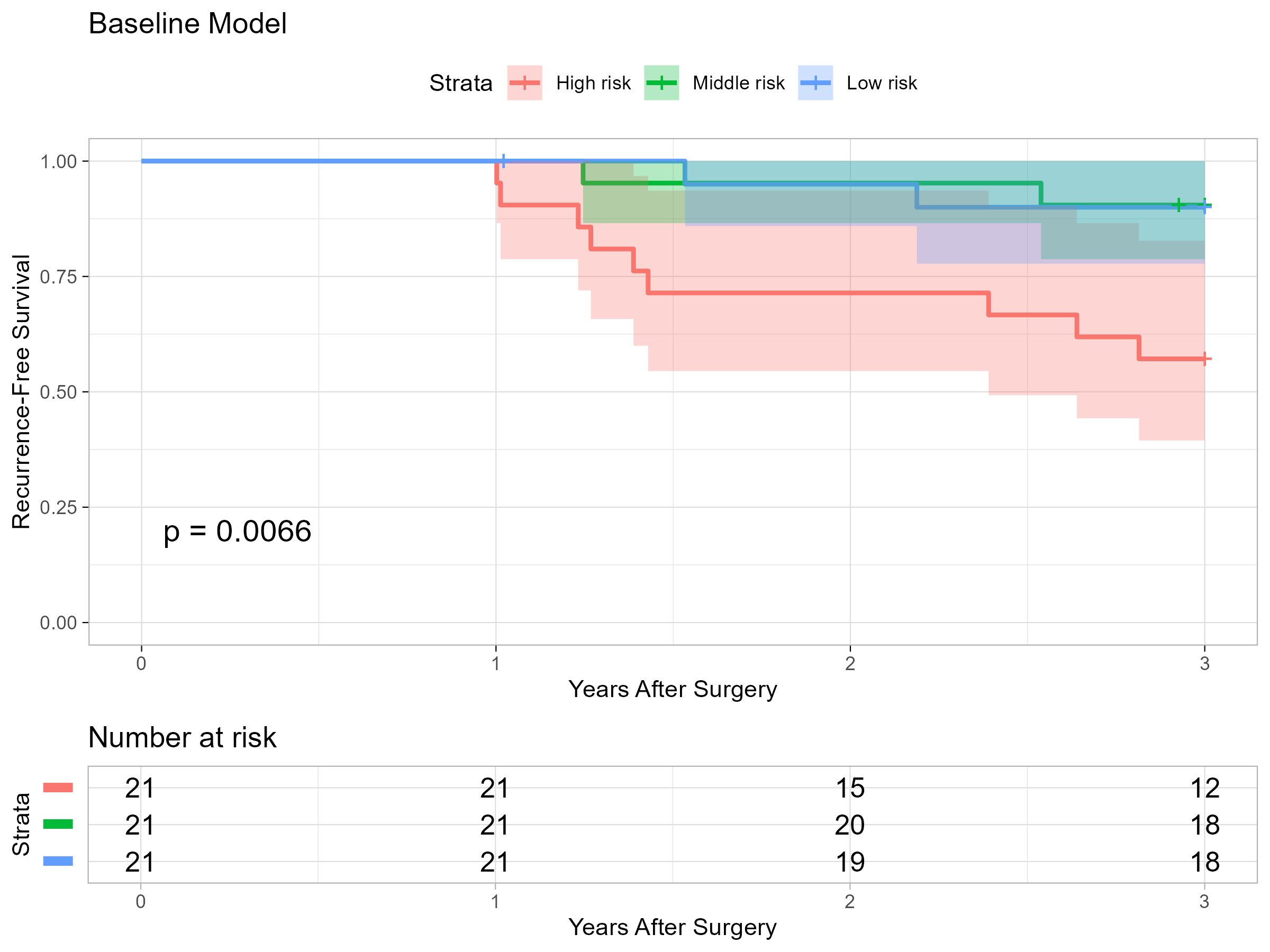
**

Figure S3 Kaplan-Meier curve of RFS stratified by event risk obtained from baseline model based on the data from 2014-01-01 to 2016-12-31.

**
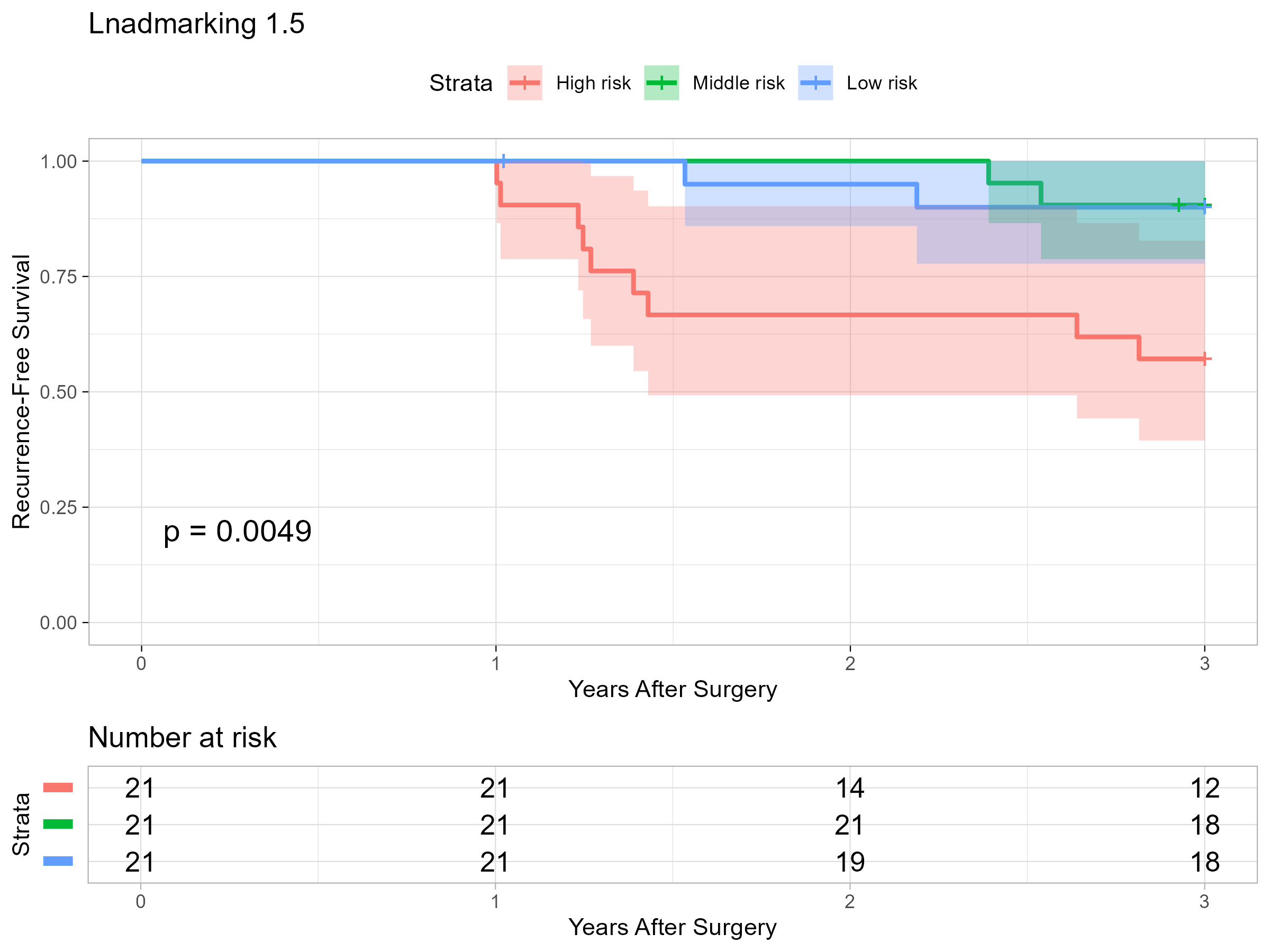
**

Figure S4 Kaplan-Meier curve of RFS stratified by event risk obtained from landmarking 1.5 based on the data from 2014-01-01 to 2016-12-31.


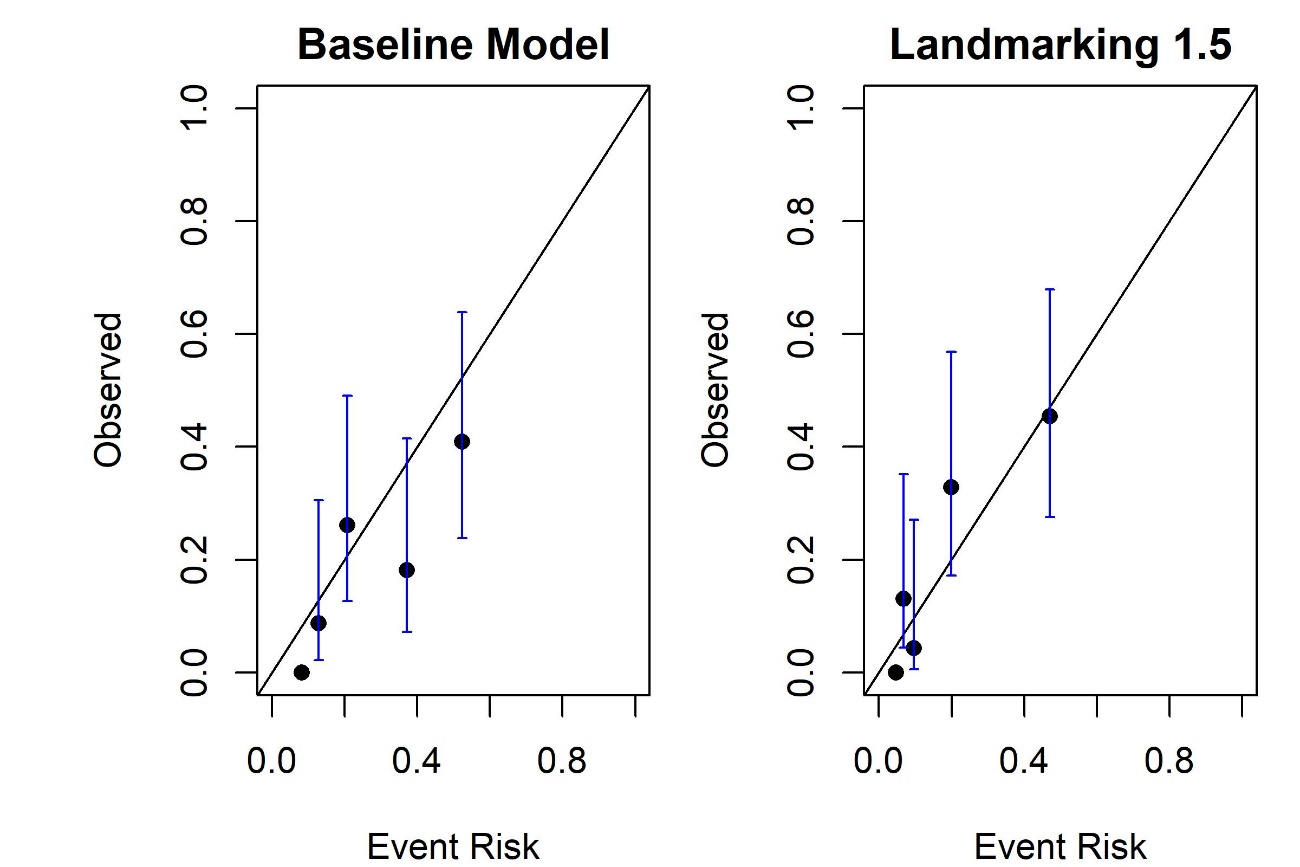


Figure S5 Calibration plot of the prediction models based on the data from 2010-01-01 to 2013-12-31.

**
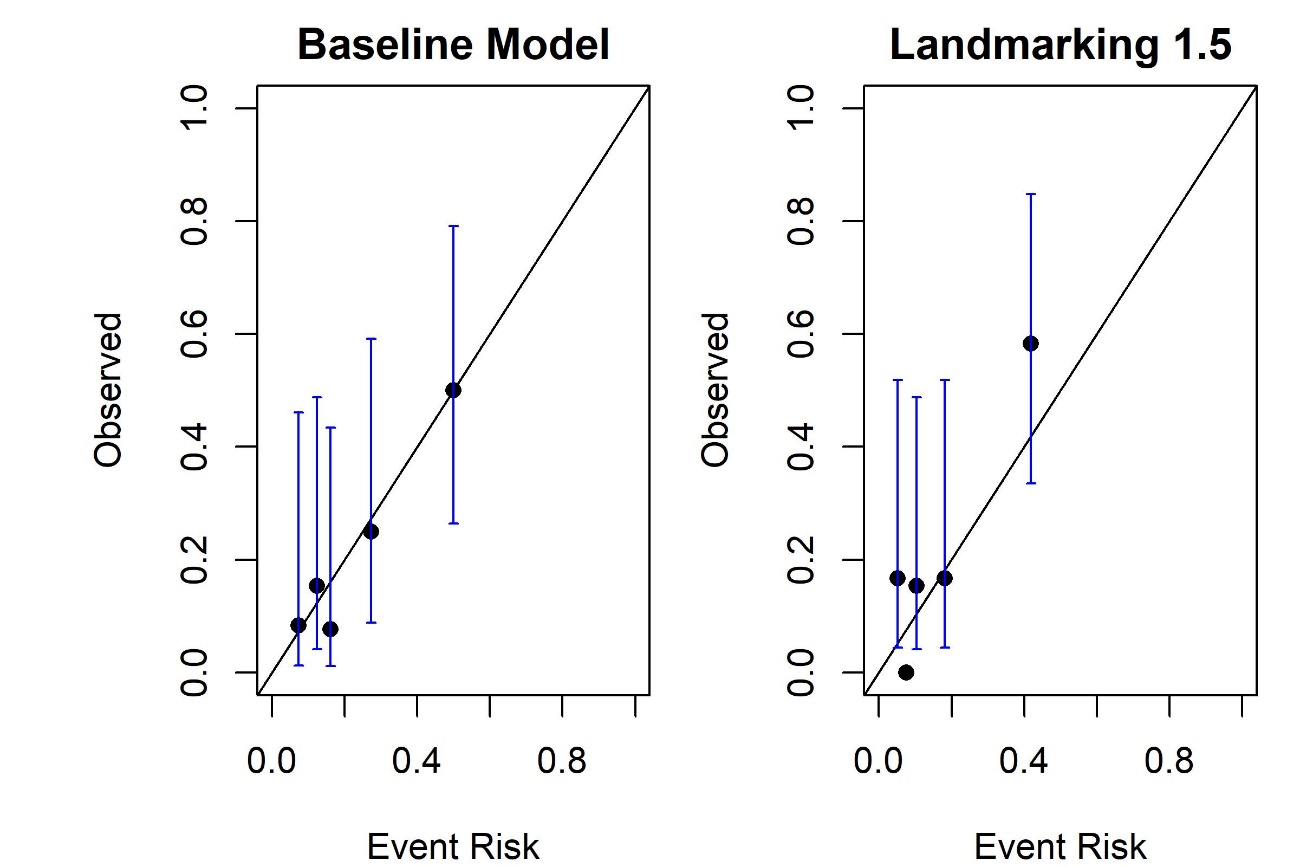
**

Figure S6 Calibration plot of the prediction models based on the data from 2014-01-01 to 2016-12-31

.
