# Supplementary material for: Development of a dynamic prediction model with the inclusion of time-dependent inflammatory biomarker enhances recurrence prediction after curative surgery for stage II or III gastric cancer
Source: Jpn J Clin Oncol. 2025 May 23;55(8):871–9. doi: 10.1093/jjco/hyaf075 (PMC12319220; doi:10.1093/jjco/hyaf075)
Supplement: Supplementary_Table2_hyaf075 [file supplementary_table2_hyaf075.doc]

Table S2 Cox regression coefficients for landmarking 1.0

|  | Effect | SE | 95% CI | |
| --- | --- | --- | --- | --- |
| blPNI | 0.264 | 0.421 | -0.561 | 1.088 |
| PNIchg | 0.203 | 0.424 | -0.628 | 1.034 |
| LVI...YES.NO | -0.192 | 1.062 | -2.273 | 1.889 |
| pT stage...T3.T12 | -0.083 | 0.619 | -1.296 | 1.131 |
| pT stage...T4.T12 | 0.278 | 0.605 | -0.907 | 1.464 |
| pN stage...N1.N0 | -0.787 | 0.770 | -2.296 | 0.722 |
| pN stage...N2.N0 | -0.413 | 0.760 | -1.902 | 1.076 |
| pN stage...N3.N0 | 1.455 | 0.548 | 0.381 | 2.529 |
| S1...6 months or more. less than 6 months | -0.162 | 0.485 | -1.113 | 0.789 |
| 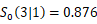 | | | | |


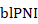
: PNI measured at baseline


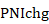
: Change in PNI from baseline

CI: confidence interval

SE: standard error


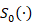
: baseline survival function
