# Supplementary material for: Development of a dynamic prediction model with the inclusion of time-dependent inflammatory biomarker enhances recurrence prediction after curative surgery for stage II or III gastric cancer
Source: Jpn J Clin Oncol. 2025 May 23;55(8):871–9. doi: 10.1093/jjco/hyaf075 (PMC12319220; doi:10.1093/jjco/hyaf075)

Table S3 Cox regression coefficients for landmarking 1.5

|  | Effect | SE | 95% CI | |
| --- | --- | --- | --- | --- |
| blPNI | 0.439 | 0.451 | -0.444 | 1.323 |
| 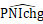 | 0.415 | 0.457 | -0.479 | 1.310 |
| LVI...YES.NO | -0.237 | 1.063 | -2.321 | 1.847 |
| pT stage...T3.T12 | -0.099 | 0.619 | -1.312 | 1.114 |
| pT stage...T4.T12 | 0.297 | 0.604 | -0.887 | 1.480 |
| pN stage...N1.N0 | -0.786 | 0.771 | -2.297 | 0.725 |
| pN stage...N2.N0 | -0.389 | 0.763 | -1.884 | 1.106 |
| pN stage...N3.N0 | 1.499 | 0.552 | 0.417 | 2.580 |
| S1...6 months or more.  less than 6 months | -0.130 | 0.489 | -1.088 | 0.829 |
| 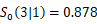  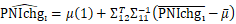  　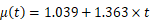  　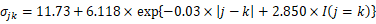 | | | | |


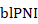
: PNI measured at baseline


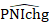
: Predicted change in PNI from baseline

CI: confidence interval

SE: standard error


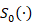
: baseline survival function


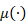
: mean of the linear model fitted to the change in PNI from baseline


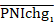
: observed history of PNI change from baseline for subject
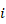
 up to before the landmark time
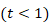
, namely
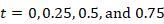


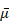
:
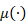
 evaluated at the observed measurement time points up to before the landmark time
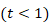


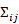
:
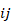
-th submatrix of the variance-covariance matrix for the change in PNI from baseline, with its
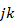
-th component givin by
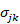

Supplement: Supplementary_Table3_hyaf075 [file supplementary_table3_hyaf075.doc]
