# Supplementary material for: Development of a dynamic prediction model with the inclusion of time-dependent inflammatory biomarker enhances recurrence prediction after curative surgery for stage II or III gastric cancer
Source: Jpn J Clin Oncol. 2025 May 23;55(8):871–9. doi: 10.1093/jjco/hyaf075 (PMC12319220; doi:10.1093/jjco/hyaf075)
Supplement: Supplementary_Table4_hyaf075 [file supplementary_table4_hyaf075.doc]

Table S4 C-statistic for temporal validation

|  | 2010-01-01 to 2013-12-31 | 2014-01-01 to 2016-12-31 |
| --- | --- | --- |
| Baseline model | 0.716 | 0.729 |
| Landmarking 1.0 | 0.782 | 0.717 |
| Landmarking 1.5 | 0.772 | 0.732 |
