# Supplementary material for: Development of a dynamic prediction model with the inclusion of time-dependent inflammatory biomarker enhances recurrence prediction after curative surgery for stage II or III gastric cancer
Source: Jpn J Clin Oncol. 2025 May 23;55(8):871–9. doi: 10.1093/jjco/hyaf075 (PMC12319220; doi:10.1093/jjco/hyaf075)
Supplement: Supplementary_Table5_hyaf075 [file supplementary_table5_hyaf075.doc]

Table S5 Reclassification table for nonevents and events based on data from 2010-01-01 to 2013-12-31

| **LM1.5**  **Baseline** | **<12.7%** | **12.7-17.4%** | **17.4-39.0%** | **≧39.0%** | **Total** |
| --- | --- | --- | --- | --- | --- |
| **<11.6%** | 26 | 0 | 1 | 0 | 27 |
| **11.6-16.7%** | 17 | 5 | 1 | 0 | 23 |
| **16.7-36.0%** | 22 | 0 | 1 | 1 | 24 |
| **≧36.0%** | 4 | 0 | 5 | 9 | 18 |
| **Total** | 69 | 5 | 8 | 10 | 92 |

Absent

| **LM1.5**  **Baseline** | **<12.7%** | **12.7-17.4%** | **17.4-39.0%** | **≧39.0%** | **Total** |
| --- | --- | --- | --- | --- | --- |
| **<11.6%** | 0 | 1 | 0 | 0 | 1 |
| **11.6-16.7%** | 3 | 1 | 1 | 0 | 5 |
| **16.7-36.0%** | 3 | 0 | 0 | 1 | 4 |
| **≧36.0%** | 0 | 0 | 2 | 9 | 11 |
| **Total** | 6 | 2 | 3 | 10 | 21 |

Present

NRI(Categorical) [95% CI]: 0.251 [ -0.063 - 0.5651 ] ; p-value: 0.11715
