# Supplementary material for: Development of a dynamic prediction model with the inclusion of time-dependent inflammatory biomarker enhances recurrence prediction after curative surgery for stage II or III gastric cancer
Source: Jpn J Clin Oncol. 2025 May 23;55(8):871–9. doi: 10.1093/jjco/hyaf075 (PMC12319220; doi:10.1093/jjco/hyaf075)
Supplement: Supplementary_Table6_hyaf075 [file supplementary_table6_hyaf075.doc]

**Table S6 Reclassification table for nonevents and events based on data from 2014-01-01 to 2016-12-31**

| **LM1.5**  **Baseline** | **<12.7%** | **12.7-17.4%** | **17.4-39.0%** | **≧39.0%** | **Total** |
| --- | --- | --- | --- | --- | --- |
| **<11.6%** | 13 | 2 | 0 | 0 | 15 |
| **11.6-16.7%** | 8 | 4 | 1 | 0 | 13 |
| **16.7-36.0%** | 3 | 7 | 1 | 2 | 13 |
| **≧36.0%** | 5 | 0 | 0 | 4 | 18 |
| **Total** | 29 | 13 | 2 | 6 | 92 |

Absent

| **LM1.5**  **Baseline** | **<12.7%** | **12.7-17.4%** | **17.4-39.0%** | **≧39.0%** | **Total** |
| --- | --- | --- | --- | --- | --- |
| **<11.6%** | 1 | 0 | 0 | 0 | 1 |
| **11.6-16.7%** | 2 | 0 | 0 | 0 | 2 |
| **16.7-36.0%** | 0 | 1 | 1 | 1 | 3 |
| **≧36.0%** | 1 | 0 | 0 | 6 | 7 |
| **Total** | 4 | 1 | 1 | 7 | 13 |

Present

NRI(Categorical) [95% CI]: 0.1292 [ -0.2327 - 0.4912 ] ; p-value: 0.48402

LM1.5: Landmarking 1.5, Baseline: Baseline model
